# Literature Review on the Utilization of Rice Husks: Focus on Application of Materials for Digital Fabrication

**DOI:** 10.3390/ma16165597

**Published:** 2023-08-12

**Authors:** Kohei Morimoto, Kazutoshi Tsuda, Daijiro Mizuno

**Affiliations:** 1 Graduate School of Design, Nagaoka Institute of Design, Niigata 9402088, Japan; 2 Center for the Possible Futures, Kyoto Institute of Technology, Kyoto 6060951, Japan; tsudakazutoshi@kit.ac.jp (K.T.); daijirom@kit.ac.jp (D.M.)

**Keywords:** green materials, rice husk, agricultural byproduct, 3D printing, digital fabrication, particleboard

## Abstract

To achieve a sustainable society, it is important to use biological resources effectively to the extent that they are renewable. Rice husk, which is abundantly produced in various regions, is a useful biomass resource. To promote their use further, it is important to expand the fields in which they are used. Therefore, this study reviews the research on rice-husk-based materials that can be used in digital fabrication, such as those used with 3D printers and Computer Numerical Control (CNC) machines, which have become increasingly popular in recent years. After outlining the characteristics of each machining method, the authors surveyed and analyzed the original research on rice-husk-based materials for 3D printers and particleboard available in digital fabrication machines for 2D machining. This review identifies issues and proposes solutions for expanding the use of rice-husk-based materials. It also indicates the need for further research on various aspects, such as the workability and maintainability of the equipment.

## 1. Introduction

In recent years, a growing body of research has focused on design strategies aimed at promoting sustainability. One prominent model proposed as a strategy for achieving circular design, as discussed in the study conducted by Moreno et al. [1], is “Design for resource conservation.” Furthermore, the butterfly system diagram put forth by the Ellen MacArthur Foundation serves as a comprehensive overview of the circular economy and delineates the following principles: “The second principle of the circular economy is to circulate products and materials at their highest value. This means keeping materials in use, either as a product or, when that can no longer be used, as components or raw materials” [2]. Hence, effective utilization of biological resources, ensuring that they are renewable, has emerged as a crucial approach for attaining sustainability.

Agricultural residues, such as rice husks from rice cultivation, can be considered major biological resources. Rice husks are an important by-product of the rice milling process and are a major agricultural waste product. Table 1 shows the statistical data on global rice paddy production released by Food and Agriculture Organization of the United Nations (FAO) [3]. Global rice production in 2020 was about 756 million tons, and rice production has increasing compared to 2010, especially in Asia and Africa. In addition to their mechanical properties, rice husks are useful materials from which its main components, cellulose, lignin, and silica, can be extracted and used as functional materials. Therefore, there have been many studies on rice husk reuse for a long time. Table 2 summarizes the number of submissions of review articles published since 2013. These results show that the number of submissions is increasing, indicating a high level of interest in rice husk utilization.

In order to promote further utilization in the future, it is important not only to develop technologies in existing areas of utilization, but also to increase utilization in new areas. One of these new areas involves the use of materials for digital fabrication. One reason is the recent development of digital fabrication technologies, such as 3D printers and laser cutters, and the other is that rice husk utilization materials have traditionally been applied in the fields of architecture and product design, where this technology is particularly actively used. The most common application in this field is to mix it with concrete and cement materials, but other research papers have been published on other materials that can be applied to construction and products, as shown in Table 3. 

Based on the above, it is expected that further research on rice husk materials that can be used in digital fabrication will promote their use in the architectural and product design fields and make even more effective use of unused resources. In fact, research papers have already been published on the use of rice husks as a material for 3D printers in 2020 and beyond. If other digital fabrication equipment, such as laser cutters and CNC machines, can also make use of this material, it will see even greater use.

Hence, this study provides a comprehensive survey of original papers focusing on the use of rice-husk-based materials for 3D printers and particleboard as a viable material for digital fabrication. By identifying recent research trends, summarizing existing research gaps, and providing recommendations for addressing these challenges, this study aims to contribute to the development of this field.

## 2. The Physical Properties of Rice Husks

Rice husk constitutes around 20% of the weight of paddy rice before milling [4]. It consists of cellulose (25–35%), hemicellulose (18–21%), lignin (26–31%), silica (15–17%), soluble matter (2–5%), and moisture (about 7.5%) [5]. In contrast to other biomass varieties, rice husk is inherently high in silica and ash content. [6]. Furthermore, due to their higher lignin content compared to wood, rice-husk-based products exhibit greater hydrophobicity compared to wood-based products [7]. The combustion of rice husks as fuel generates rice husk ash as a by-product, constituting about 20–25% of the weight of rice husks [8]. Generally, rice husk ash contains approximately 85–95% amorphous silica, but the physical properties and characteristics of rice husk ash depend on the processing parameters, including combustion methods, separation processes, and milling [9]. Materials derived from rice husk ash are characterized by their porous nature and are used as adsorbents and fillers.

## 3. Targeted Digital Fabrication Machines

Digital fabrication machine is a generic term for machine tools that process materials based on computer-designed data; a typical example is the 3D printer.

**Figure 1:**
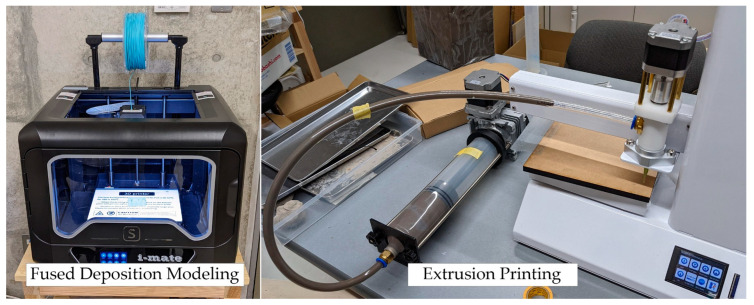
3D printer (fused deposition modeling type and extrusion printing type).

Three-dimensional (3D) printing technology, also called additive manufacturing, is a method of layering materials based on data created by 3D computer-aided design (3D CAD) or 3D data generated by a 3D scanner. Various layering methods exist, including applying thermally melted resin or paste materials layer-by-layer, curing liquid resins with ultraviolet light, and sintering powdered materials. Two typical types of machine are shown in Figure 1.

**Figure 2:**
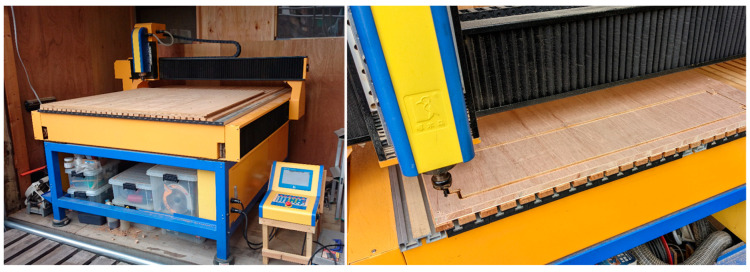
Computer numerical control (CNC) machine.

In addition to 3D printers, digital fabrication also uses equipment such as computer numerical control (CNC) machines and laser cutters that can cut board materials and other flat materials based on 2D data created with CAD or Graphic Soft. A CNC milling machine is a tool that cuts materials according to 2D data using an end mill. The equipment is shown in Figure 2.

**Figure 3:**
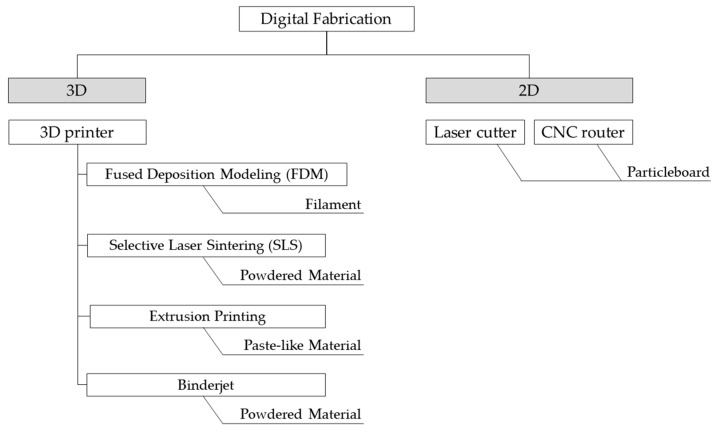
The target machines and materials in this article.

This tool can also utilize 3D data to perform 3D cutting. It is also known as “subtractive manufacturing” because it removes unnecessary parts from the materials. A laser cutter is a machine tool that cuts or engraves objects using a laser beam instead of an end mill. Both technologies are expected to be utilized in the fields of architecture and product design. Therefore, this section introduces research on materials utilizing rice husks for 3D printers and board materials that can be used in 2D processing. The target machines and materials are shown in Figure 3.

## 4. Articles on Materials for 3D Printer Utilizing Agricultural Wastes Including Rice Husks

### 4.1. Method

Searches on ScienceDirect and Springer only produced one review article and three research articles on the utilization of rice husk as a material for 3D printers. Therefore, in this section, Google Scholar was additionally used to search for the keyword “rice husk 3D printer”. We extract relevant articles from 2013 to 2022 that contained the keyword in the title and extracted relevant articles after reviewing the abstracts. Among them, we select only those articles that mentioned studies utilizing rice husks as a raw material. After categorizing them into review articles and original articles, we will provide an overview of each and mention some important issues.

### 4.2. Review Articles on Materials for 3D Printer Utilizing Agricultural Wastes Including Rice Husks

Among the various 3D printing methods, fused deposition modeling (FDM) is the most popular method, in which a string of thermoplastic polymer filaments is melted and layered with heat. Polylactic acid (PLA) and acrylonitrile butadiene styrene (ABS) are typical materials used in FDM. Numerous studies have been reported on blending natural fibers with these materials to produce composite filaments, and several review articles have been published. Five review articles were extracted as a result of the survey using the above methods. 

Most recently, Rajendran et al. summarized an approach using natural-fiber-reinforced polymer composites as filaments for 3D printers. They analyzed the effects of fiber treatment, material preparation methods, and compatibilizer additions and reviewed methods for producing filaments from composites [10]. Ahmed et al. also summarized a study on the properties of polymers in 3D printing filaments with added natural fibers [11]. They noted that in many studies, the average particle size of the fiber material used as filler was described, but detailed fiber structure information was lacking. They suggested that it is important to carefully analyze the quality and composition of the filaments before printing. Mazzanti et al. noted that further investigations on other semicrystalline polymers would be interesting, noting that materials less common in FDM, such as polyethylene (PE) and polypropylene (PP), can benefit from composites with natural fibers and particles [12]. The review also specifically focused on studies using PLA as a matrix, discussing the properties of PLA-based bio-composites and printing requirements, as well as future opportunities for applications, upcycling and recycling, and biorefineries [13]; Seker et al. proposed a method for manufacturing acoustic panels using additive manufacturing and presented the acoustic properties of composites reinforced with natural fibers, noting that small voids form between the deposition lines in the 3D-printed composites. The authors concluded that these voids provided an advantage for the acoustic panels to effectively absorb sound [14].

As mentioned above, there are several review articles on 3D printing filaments with added natural fibers. However, these articles mainly focus on FDM 3D printers, and there are no papers that organize material studies for other 3D printers. Therefore, this paper will research papers on the application of rice husks as a material for various 3D printing methods, including FDM.

### 4.3. Original Articles on 3D Printer Materials Using Rice Husks

Examples of research articles on 3D printer materials using rice husks are listed in Table 4. For each article, the method, matrix, and filler or binder materials are provided.

#### 4.3.1. Fused Deposition Modeling

**Figure 4:**
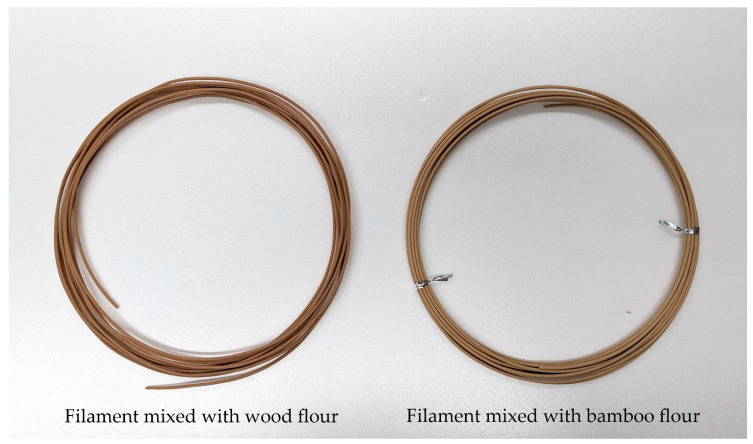
Filaments mixed with natural fiber materials.

The most common way to utilize natural resources as a material for 3D printers is to mix them with a polymer as a matrix to generate a composite filament. As an example, filaments mixed with wood and bamboo powders are shown in Figure 4.

This method is also the most common way to apply rice husks as a 3D printer material, and various studies have been conducted to improve material performance and modeling quality. For example, Tsou et al. added methylene diphenyl diisocyanate as an interfacial compatibilizer to improve the tensile strength and impact resistance (Izod impact) of composite materials. They also observed that a porous morphology was generated in the composite material and that the pore size increased considerably with the rice husk content [15] Contrastingly, Wu et al. fabricated filaments with acrylic acid-grafted PLA (PLA-g-AA) and coupling-agent-treated rice husks to improve the properties of bio-composites. They reported that the developed material achieved improved tensile properties and water resistance [16]. le Guen et al. produced 3D-printed filaments by blending wood and rice husk powders with PLA and analyzed their properties. They observed differences in the visual evaluation and process stability, attributed it to the silica content and particle size in the powders, and considered that the particle size of the materials may have a considerable influence on their rheological behavior [17].

There have also been studies that used materials other than PLA as the base material. Morales et al. studied rice husks and filaments using recycled polypropylene (rPP). rPP with rice husk fibers has some problems, such as increased water absorption and swelling diameter and faster degradation than rPP alone; however, it can reduce the warpage caused by the printing process and can be used in applications where its mechanical properties are acceptable [18]. Research on blends of rice husk ash and ABS was conducted, noting that a powder mixture of ABS and rice husk ash is highly useful in the manufacture of optical components by 3D printing because of its ability to increase the refractive index and decrease the absorption coefficient [19].

#### 4.3.2. Selective Laser Sintering (SLS)

The SLS method is a 3D printing technology in which a powder bed is selectively scanned by a laser and processed during layer-by-layer sintering. It is characterized by low material loss and the ability to create highly accurate models. Polycarbonate and polystyrene powders are the commonly used materials. Although there are not many examples of the utilization of rice husks using this method, a method of fabricating composites for SLS by adding rice husk powder to CO-polyamide powder, which is utilized as a hot melt adhesive, has been proposed [20]. This study confirmed the effect of rice husk content on the mechanical properties, dimensional accuracy, and residual ash content of parts, as well as the sintering properties of the material, and clarified the requirements for optimizing the SLS 3D printer method for investment casting parts, which are frequently applied in many cases.

#### 4.3.3. Extrusion Printing

Extrusion printing is a 3D printing method in which a paste-like material is injected into a cylinder and extruded through a nozzle using a screw or air pressure. The most well-known example of this technique is the use of a large 3D printer to produce cement to form architectural structures. Food 3D printers, which have been actively developed in recent years, are also based on this method. In this method, the rheological properties of the paste material to be extruded, as well as the means and time required for curing after printing, are major issues.

The building industry has seen an increase in the research and use of 3D printing, and research has been conducted on the 3D printing of cement using rice husk ash. Muthukrishnan fabricated a rectangular structure with rice husk ash added, utilizing a screw-type extruder, the same system used for 3D printing in architecture, and evaluated the properties of the structure against bare mortar [21]. He then considered that the structure to which rice husk ash was added showed less shape change over time, which was attributed to its faster curing in the mortar and suppressed shape deformation owing to self-loading. Therefore, he concluded that the addition of rice husk ash improved the model. Palaniappan et al. detailed the potential of 3D printing in the construction industry in their study utilizing waste materials, such as cow dung ash, rice husk ash, sugarcane bagasse ash, ground granulated blast-furnace slag (GGBS), and marble dust as concrete materials [22]. Research is also being conducted on the application of rice husks as a material for the 3D printer architecture of soil structures. This is based on the traditional building technique called cob, which is a method for building structures by layering moist clayey soil. The Italian 3D printer company WASP produces a 3D printer for soil structures. The material used was a composite of soil mixed with rice husks and lime. The contact surface between the rice husks and the soil matrix increases, promoting the formation of Si-O-Si bonds and improving the stiffness of the material [23]. Nida et al. investigated the effect of the addition of guar gum (GG) to ground rice hulls of different particle sizes, with and without the addition of GG, on printability. The ratio of powdered rice hulls to guar gum was determined, and its application in food packaging was proposed [24]. A case study of in situ mullite (3Al_2_O_3_-2SiO_2_) foam development using silica extracted from rice husk ash was investigated. Al_2_O_3_-SiO_2_ ink was prepared using an aqueous binder containing α-alumina and two types of silica (rice-husk-ash-extracted biogenic nano-silica and commercial silica). The material was extruded by air pressure through a conical nozzle, dried in an air furnace, and fired in a muffle furnace. Its application to high-performance products (e.g., insulators, filters, and high-temperature operating devices such as burners) was proposed [25].

#### 4.3.4. Binder Jetting

The binder jetting method produces a three-dimensional model object by repeatedly placing the powdered material on a bed and injecting a liquid binder in layers. As with SLS, there is little loss of material; however, there is a problem in that the strength after modeling is low, and post-processing is sometimes required. For example, Zeidler et al. applied lignin sulfonate, sodium silicate (water glass), and polyvinyl alcohol as binders to Miscanthus sinensis particles, wood flour, shell powder, and fruit stone powder; evaluated the properties of the moldings; and discussed the optimal combinations. Miscanthus/PVA was proposed as an appropriate combination, and it was also confirmed that spray coating with a sodium silicate solution can improve the mechanical strength and water resistance. In contrast, they mentioned that the mechanical strength was weaker than that of polymer-based components, making them suitable for applications where they are not subjected to loads, and suggested their application in food packaging. The report mentioned that rice husks were not included because no considerable results have been achieved; however, they are interesting to investigate because they are abundantly available in many regions and are expected to be developed in the future [26].

## 5. Articles on Materials for Particleboard Using Rice Husks

Particleboard is a material typically produced by hot-pressing wood chips sprayed with an adhesive that serves as a binder. An intermediate layer composed of coarse particles and fibers is sandwiched between layers of fine particles to achieve strength and a light weight. However, obtaining the wood chips used as raw materials is fraught with deforestation and raw material shortage problems. Urea-formaldehyde, which is mainly used as an adhesive, has been pointed out to be harmful to the human body. Particleboards can also be processed by CNC or laser cutters and are widely used as building and furniture materials, along with veneer boards. Agricultural waste would be an effective means of resource recycling and would help stabilize the supply of board materials if it could be utilized as raw materials for these boards.

This study surveys research articles on the development of particleboards utilizing rice husks, as there are currently no studies on the use of rice husks in digital fabrication. 

Original articles were searched using the keywords “rice husk” and “particleboard” on the ScienceDirect and Springer websites, and all keywords must be included in the title. Table 5 shows the extracted articles organized by material/binder/characteristic treatment.

There are many examples of research on particleboards made from rice hulls, in which different combinations of materials and binders were used to evaluate mechanical strength, water resistance, and heat resistance. Olupot et al. found that the addition of sawdust improved the properties of rice-husk-based, low-density particleboard. They utilized commercially available synthetic adhesives as binders instead of the commonly used urea-formaldehyde and phenol-formaldehyde for particleboard manufacturing, with promising results [27]. Akinyemi et al. processed agricultural waste, peanut shells, and rice husks into particles and used them to produce composite panelboards. In addition, cassava starch blended with urea-formaldehyde was used as an adhesive to reduce the environmental impact, presenting its potential as an eco-friendly interior panelboard material [28]. Huang et al. evaluated the feasibility of manufacturing particleboard by combining insect breeding residues and rice husks in different ratios and using citric acid/tapioca starch as a natural binder. Although a decrease in the rupture factor was observed as the percentage of insect breeding residue increased, they identified a combination ratio of materials that met the Japanese Industrial Standard (JIS) specifications, showing its potential as a board material [29].

Some studies have fabricated and characterized fiberboard and particleboard using hemp staple fibers and rice husk particles, respectively. Cornstarch was employed as the binder, and the boards were prepared by hot-pressing the binder-impregnated material. Fiberboard is stiffer than particleboard and has been proven to be able to withstand greater loads, meeting standards that allow it to be recommended as an interior material [30]. The same material and binder combination is also used as a fire retardant, which is a challenge when used as a building material. Ammonium dihydrogen phosphate was added as a flame retardant, and boards were manufactured to find the optimal combination and meet the high flame-retardant standards [31].

As confirmed from the above studies, it is difficult to guarantee the strength particleboards containing rice hulls, which have a smaller aspect ratio compared common particleboards; and, when naturally derived adhesives are used, there is a high possibility that various types of performance will be degraded. Therefore, some attempts have been made to improve performance with additional treatments. For example, Nicolao et al. studied how to improve the function of manufactured rice husk boards by utilizing soy protein as a binder. They achieved functional improvements, including water resistance, by impregnating the manufactured boards with tung oil [32]. Chalapud et al. also fabricated boards using the same binder. They achieved improved mechanical strength by sandwiching the surface of the board between jute cloth [33]. Another method to increase the mechanical strength through sandwich construction is to build wood strand layers on the surface. The binder was a common formaldehyde adhesive; however, it was a good substitute for wood strand boards [34].

Finally, a special case is the manufacturing method of layer-by-layer treatment [35]. In this method, a binder is coated on the surface of the rice husks by vapor deposition and then hot-pressed to produce the board. Strong mechanical properties were obtained due to structures bonded via strong electrostatic interactions that occurred at the molecular scale. Furthermore, the polymer system used branched polyethyleneimine and polyacrylic acid, and layer-by-layer approaches have environmentally friendly features, such as the use of water as a solvent and optimal performance at room temperature and low solution concentrations. Such an efficient surface modification method has shown promising sustainable potential.

## 6. Discussion

This study reviewed the literature on the effective use of rice husks, focusing on their utilization in the fields of architecture and product design. The results of the survey of review articles on the use of rice husks showed a high number of review articles on the use of incinerated ash as a concrete filler, indicating a high level of interest. They have been published continuously since 2017 and the number is still increasing, suggesting that this method is a promising means of incinerated ash utilization. In addition, it was confirmed that rice husks have the potential for a wide variety of applications. In many cases, husks are utilized as a base material or filler or the silica and lignin components of the husks are extracted and utilized. In deciding which method to choose, the performance of the rice-husk-derived materials and their economics and the balance between the yield of each region and the amount of treatment with each utilization method are important guidelines. Therefore, it will be necessary in the future to analyze the amount of rice husks processed and the cost aspects of each utilization method.

The most common method when using rice husks as a material for 3D printers is to mix them as a filler in filaments used for FDM 3D printers, which are the most widespread type of 3D printer. PLA was the most common polymer used in these studies as the base polymer for mixing rice husks. In general, adding natural fiber fillers reduces the mechanical strength and durability; hence, efforts have been made to compensate for these problems. However, additives are often included to overcome these problems, causing costs to increase. In the future, it will be necessary to consider both the purpose and the cost of the material. While it was noted that there was insufficient information on particle size in each of the studies, other studies indicated that the particle size of the material could have a significant impact on its rheological behavior. Therefore, it is important in the future to establish a method for measuring filler particle size and to unify this information.

Rice husks also have the potential to be used as an additive to materials, especially in cement extrusion printing. Because the addition of rice husks leads to retention of the shape of the modeled object until hardening, it is very effective as a material for 3D printing without using a mold. Although it is still a developing technology, rice husks have been used as materials for concrete in the past and their usage is expected to expand along with the spread of 3D printing in construction.

The possibility of using rice husks as materials for SLS has been confirmed. However, the powder materials used in this method tends to scatter easily, affecting the installation environment of the equipment. If rice husks are used, their particle size and specific gravity should be compared with those of ordinary nylon materials to evaluate their ease of handling, equipment cleanliness, and work environment.

Extrusion printing is a useful method for a wide range of applications from architecture to the manufacture of functional products. However, the material in this method is wet, and the state of the material changes with time until modeling. Material drying always occurs using this method. In addition, when adding particles, such as rice husks, material separation may occur depending on the rheological properties of the binder. Furthermore, when mixing organic materials, excess material and residue in the cylinder can cause mold and corrosion. In the case of rice husk ash, the risk is low. However, if the rice husk powder is used directly, this issue should be considered. Based on the above, to expand the use of rice husks in this method, it is necessary to obtain knowledge on the speed of aging and deterioration of the physical properties of the material before firing or hardening. It is also important to develop equipment that is easy to clean.

The use of rice husks in particleboards has been studied, regardless of the digital fabrication trend. All of these particleboards tend to have reduced mechanical properties compared to common wood particleboards, and the focus is on how to reduce performance loss and maintain this within the standard. These studies have focused on improving properties such as mechanical strength and have not mentioned the workability of the material. In particular, there is no research on the machinability of CNC or laser cutters for these materials. Therefore, to promote the use of rice-husk-derived particleboard as a material for digital fabrication, it is necessary to evaluate its workability.

## 7. Conclusions

The following is a summary of the issues and solutions associated with the use of rice husks as materials for digital fabrication.

- Applications suited to material properties

Mixing rice husk fibers with neat materials, such as polymer composites, tends to lead to performance degradation. However, the addition of other materials or treatments to improve functionality can increase the costs. Therefore, it is important not only to aim for improved functionality, but to also consider applications that match the characteristics of the material.

- Evaluation of actual operation (long-term quality of materials/maintainability of equipment)

The paste used in the material extrusion method tends to change over time, and the material remaining in the syringe or at the nozzle tip can affect the printing characteristics. However, previous studies have not considered the aging of these materials or operational concerns. Therefore, future studies should investigate the changes in the material properties over time and consider the maintainability of the equipment.

- Processability of board materials

When rice-husk-derived particleboard is cut by CNC, the silica component in the material may shorten the life of the end mills. Depending on the internal density and interparticle adhesion strength, the material on the cut surface may peel off when milled. If the material peels off during processing by CNC, it may be improved by reducing the grain size or increasing the adhesive strength. However, the amount of silica cannot be changed significantly; therefore, when machining with CNC, it is effective to deal with this problem by slowing down the machining speed or using end mills for metal rather than wood. When processing with a laser cutter, it is also necessary to verify the reaction of the rice husk components with the laser beam. Therefore, to utilize a rice-husk-based particleboards for digital fabrication, research on their processability and optimal processing conditions would help expand their use.

Despite the abovementioned issues, the use of rice husks as a structural material for buildings and industrial products is an effective method of material recycling because it leads to mass disposal of agricultural waste and long-term carbon fixation.

This study describes the research trend on the use of rice husks for digital fabrication and discussed the possibilities of rice husk use, as well as issues and solutions. The promotion of material research for digital fabrication, which is expected to be further utilized in the future, will lead to further effective utilization of agricultural waste. This review will help to expand the use of rice husks in the architectural and product design fields.

## Figures and Tables

**Table 1:** Annual production quantity of rice paddies in 2010 and 2020 (FAOSTAT).

Region	Production Quantity [×10^6^ton]	
	2010	2020
World	694.5	756.7
Africa	26.0	37.9
Americas	36.5	38.1
Asia	627.5	676.6
Europe	4.3	4.1
Oceania	0.2	0.1

**Table 2:** Number of review articles published on rice husks (ScienceDirect). A keyword search for “rice husk” was performed in ScienceDirect, and the results were sorted by checking the “Review Article” checkbox. The results for 2013–2022 are listed and categorized into those where the research content and application methods were analyzed broadly and those where a more detailed review was conducted by narrowing down the application cases.

Research Target		2013	2014	2015	2016	2017	2018	2019	2020	2021	2022	Total
General	Rice husk	0	0	1	0	0	0	0	1	0	1	3
	Rice husk ash	0	1	0	1	0	0	1	0	0	0	3
	Rice husk derived silica	0	0	0	0	1	0	0	0	0	0	1
Application	Cement/concrete	1	0	0	0	2	2	1	3	4	2	15
	Adsorbents, soil conditioners, fertilizers	0	0	0	0	0	1	2	0	2	0	5
	Energy	0	0	1	2	1	0	0	0	0	2	6
	Fiber-reinforced polymer composites	0	0	0	0	0	0	0	0	1	0	1
	Application to metal matrix	0	0	0	0	0	0	0	0	0	1	1
	Application to alkali activated materials	0	0	0	0	0	0	0	0	0	1	1
	Total	1	1	2	3	4	3	4	4	7	7	36

**Table 3:** Number of research articles published on the application of rice husks that can be used in the construction and product fields (ScienceDirect and Springer). Keyword searches for “rice husk” were conducted on Elsevier’s ScienceDirect and Springer’s article search sites, and the results from 2013 to 2022 that included the keyword in the title were listed. A total of 1243 search results were found in ScienceDirect and 535 in Springer. The titles of each article were then checked, and the articles that corresponded to the application items in Table 3 were extracted and classified.

Application	2013	2014	2015	2016	2017	2018	2019	2020	2021	2022	Total
Polymer composite	2	5	3	5	8	5	10	16	21	14	89
Metal matrix composite	3	1	2	0	1	3	2	2	1	4	19
Rubber composite	1	0	0	1	0	1	1	2	4	2	12
Glass	1	0	2	0	1	0	3	1	0	2	10
Particleboard	1	0	0	0	1	2	0	2	0	3	9
Building materials (board/panel)	0	0	0	1	0	1	1	3	1	0	7
3D printing materials	0	0	0	0	0	0	0	1	1	1	3
Others	1	0	1	1	0	0	0	1	1	6	11
	9	6	8	8	11	12	17	28	29	32	160

**Table 4:** Original articles on materials for 3D printers using rice husks.

3D Printing Method	Matrix	Filler or Binder	Authors	Year
FDM	PLA	Rice husk	Tsou et al. [15]	2019
FDM	PLA	Rice husk	Wu and Tsou [16]	2019
FDM	PLA	Rice husk/wood	le Guen et al. [17]	2019
FDM	Recycled polypropylene	Rice husk	Morales et al. [18]	2021
FDM	ABS	Rice husk ash	Peng et al. [19]	2021
SLS	Co-polyamide	Rice husk	Li et al. [20]	2022
Material Extrusion	Cement	Rice husk ash	Muthukrishnan et al. [21]	2022
Material Extrusion	Cement	Rice husk ash	Palaniappan [22]	2020
Material Extrusion	Soil	Rice husk and lime	Ferretti et al. [23]	2022
Material Extrusion	Rice husk	Guar gum (as a binder)	Nida et al. [24]	2021
Material Extrusion	Al_2_O_3_-SiO_2_ ink (includes rice-husk-derived silica)	PVA solution, glycerol, and dispersant (as a binder)	Hossain et al. [25]	2022
Binder jetting	Miscanthus particles, wood flour, seashell powder, fruit stone flour, rice husk (only mentioned)	Lignin sulfonate/sodium silicate/polyvinyl alcohol (as a binder)	Zeidler et al. [26]	2018

**Table 5:** Original article on particleboard utilizing rice husks.

Matrix	Binder	Treatment	Authors	Year
Rice husks/sawdust	Synthetic adhesives Fevicol/ Ponal/Woodfix		Olupot et al. [27]	2022
Mixture of groundnut shell and rice husk particles	Cassava starch blended with urea-formaldehyde		Akinyemi et al. [28]	2022
Mixture of insect rearing residue and rice particles	Citric acid/tapioca starch		Huang et al. [29]	2022
Rice husk particles/ hemp fibers	Cornstarch		Battegazzore et al. [30]	2018
Rice husk particles/ hemp fibers	Cornstarch	Add ammonium dihydrogen phosphate to the binder	Battegazzore et al. [31]	2018
Rice husk particles	Soy protein concentrate	Impregnate the board with tung oil	Nicolao et al. [32]	2020
Rice husk particles	Soy protein concentrate	Add two jute fabric layers to the board	Chalapud et al. [33]	2020
Rice husk particles	Phenol-formaldehyde resin	Sandwiched between two wood strand layers	Kwon et al. [34]	2013
Rice husk particles	Branched polyethylene imine and poly acrylic acid	Layer by Layer-coating on rice husks	Battegazzore et al. [35]	2017

## Data Availability

All the data are available in the manuscript.
